# Polo-like kinase-1, Aurora kinase A and WEE1 kinase are promising druggable targets in CML cells displaying BCR::ABL1-independent resistance to tyrosine kinase inhibitors

**DOI:** 10.3389/fonc.2022.901132

**Published:** 2022-08-05

**Authors:** Manuela Mancini, Sara De Santis, Cecilia Monaldi, Fausto Castagnetti, Annalisa Lonetti, Samantha Bruno, Elisa Dan, Barbara Sinigaglia, Gianantonio Rosti, Michele Cavo, Gabriele Gugliotta, Simona Soverini

**Affiliations:** ^1^ IRCCS Azienda Ospedaliero-Universitaria di Bologna, Istituto di Ematologia “Seràgnoli”, Bologna, Italy; ^2^ Dipartimento di Medicina Specialistica, Diagnostica e Sperimentale Università di Bologna, Bologna, Italy; ^3^ Department of Biomedical and Neuromotor Sciences, University of Bologna, Bologna, Italy

**Keywords:** chronic myeloid leukemia, imatinib resistance, polo like kinase-1, aurora kinase A, WEE1 kinase

## Abstract

In chronic myeloid leukemia (CML), Aurora kinase A and Polo like kinase 1 (PLK1), two serine-threonine kinases involved in the maintenance of genomic stability by preserving a functional G2/M checkpoint, have been implicated in BCR::ABL1-independent resistance to the tyrosine kinase inhibitor (TKI) imatinib mesylate and in leukemic stem cell (LSC) persistence. It can be speculated that the observed deregulated activity of Aurora A and Plk1 enhances DNA damage, promoting the occurrence of additional genomic alterations contributing to TKI resistance and ultimately driving progression from chronic phase to blast crisis (BC). In this study, we propose a new therapeutic strategy based on the combination of Aurora kinase A or PLK1 inhibition with danusertib or volasertib, respectively, and WEE1 inhibition with AZD1775. Danusertib and volasertib used as single drugs induced apoptosis and G2/M-phase arrest, associated with accumulation of phospho-WEE1. Subsequent addition of the WEE1 inhibitor AZD1775 in combination significantly enhanced the induction of apoptotic cell death in TKI-sensitive and -resistant cell lines as compared to both danusertib and volasertib alone and to the simultaneous combination. This schedule indeed induced a significant increase of the DNA double-strand break marker γH2AX, forcing the cells through successive replication cycles ultimately resulting in apoptosis. Finally, combination of danusertib or volasertib+AZD1775 significantly reduced the clonogenic potential of CD34+ CML progenitors from BC patients. Our results may have implications for the development of innovative therapeutic approaches aimed to improve the outcomes of patients with multi-TKI-resistant or BC CML.

## Introduction

Chronic myeloid leukemia (CML) is characterized by the Philadelphia (Ph) chromosome resulting from the t(9;22)(q34;q11) translocation that generates the *BCR::ABL1* oncogene. *BCR::ABL1* encodes a chimeric protein that shows constitutive tyrosine kinase (TK) activity and drives clonal expansion of leukemic hematopoiesis (1). Over the past two decades, targeted therapy with TK inhibitors (TKIs) (imatinib [IM], nilotinib, dasatinib, bosutinib or ponatinib) has shown excellent results. However, the development of resistance remains a critical problem for the management of CML (2, 3). Primary and secondary resistance to TKIs may be triggered by BCR::ABL1-independent signals potentially able to induce the self-maintenance of the leukemic stem cell (LSC) clone (4–12). In these cells, mitogen-activated protein kinase (MAPK), phosphoinositide 3-kinase (PI3K), Hedgehog (Hh), WNT, the polycomb gene BMI1 and Notch remain active despite BCR::ABL1 kinase inhibition (12–15). Hence, there is a great interest in the characterization of signals underlying BCR::ABL1-independent resistance, in an attempt to develop novel eradicating strategies.

We have previously shown that the hyper-activation of Aurora A and Polo like kinase 1 (PLK1) serine/threonine kinases and of FOXM1 transcription factor plays a crucial role in maintenance and survival of CD34+ progenitors and in BCR::ABL1+ LSC persistence under TKI therapy (16). In this study, we extend our investigations to Aurora A and PLK1 inhibition, that is already used for clinical purposes in acute myeloid leukemia and myelodysplasias (17), starting from the evidence that the integrity of signaling pathways responsible for cell cycle arrest, remodeling of chromatin and repair of damaged DNA are critical to ensure replication fidelity.

While normal cells repair DNA damage upon G1 arrest, leukemic cells often display a deficient G1/S checkpoint, thus requiring a functional G2/M checkpoint for efficient DNA repair. Aurora kinase A plays a key role in the centrosome cycle and polar spindle assembly checkpoint, ensuring regulated progression from G2 to M and throughout the M phase. Hence, it is not surprising that Aurora A is frequently overexpressed in cancers, which correlates with a poor prognosis (18–20). PLK1 plays an equally essential role in the regulation of cell division and maintenance of genome stability in mitosis, in spindle assembly, and in DNA damage response (21–26). Active PLK1 can phosphorylate WEE1, an oncogenic nuclear tyrosine kinase that negatively regulates cyclin B-CDK1 complexes, thus controlling cell cycle progression at the crucial G2/M checkpoint. Therefore, WEE1 represents a key player in the DNA damage repair process and acts to prevent mitotic entry in cells harboring damaged DNA. Overexpression of WEE1 has been identified in several malignant conditions including leukemias (27).

Aurora kinase A and PLK1 are involved in regulation of mitotic entry by controlling cyclin B1-CDK1 activity. PLK1 promotes the recruitment of Aurora kinase A to the centrosomes in G2, which, in turn, induces Aurora kinase A-mediated recruitment of cyclin B1. PLK1 activates cyclin B1-CDK1 complexes *via* degradation of the CDK-inhibitory kinase WEE1 (28). Aurora kinase A and PLK1 overexpression may thus be associated with genomic instability, one major trait of CML LSCs. Our hypothesis is that, in CML, Aurora kinase A together with PLK1 may cooperate with the constitutively activated BCR::ABL1 tyrosine kinase promoting the occurrence of additional genetic and genomic alterations that may be direct or indirect drivers of TKIs resistance and disease progression to blast crisis (BC). In this study, we evaluated a new therapeutic strategy based on Aurora kinase A or PLK1 inhibition with PHA-739358 (danusertib) or BI6727 (volasertib), respectively, followed by WEE1 inhibition with AZD1775. This strategy was found to be effective in cell lines displaying BCR::ABL1-independent TKI resistance and in primary cells from CML patients in BC.

## Materials and methods

### Materials

Imatinib, danusertib, volasertib and AZD1775 were purchased from Selleck Chemicals. For Western blotting, 10% acrylamide gels, running buffer (MOPS), transfer buffer and polyvinylidene difluoride (PVDF) transfer membrane were bought from Thermo Scientific. For immunofluorescence (IF), the FITC-conjugated anti-mouse IgG, the anti-rabbit conjugated with Alexa Fluor 568 antibodies and DAPI (6-diamidino-2-phenylindole) were purchased from Sigma-Aldrich.

The anti-cleaved-caspase 3 (Asp175), anti-cleaved-caspase 9 (Asp353), anti-BAX, anti-CHK1, anti-phospho-CHK1 (S317), anti-CHK2, anti-phospho-CHK2 (T68), anti-cyclin B1, anti-phospho-cyclin B1 (S133), anti CDC25C, anti-phospho-CDC25C (S198), anti-WEE1, anti-phospho-WEE1 (S642), anti-CDK1, anti-phospho-CDK1 (Y15), anti-phospho-H2AX (S139), anti-RAD51, anti- β-tubulin Alexa Fluor 555 Conjugate, anti-Aurora kinase A, anti-phospho-Aurora kinase A (T288), anti-PLK1, anti-phospho-PLK1 (T210), anti-PARP, anti-caspase-3 and anti-caspase-9 antibodies were purchased from Cell Signaling Technology. The anti-β-actin antibody used as loading control was purchased from Santa Cruz Biotechnology.

### Cell lines, patients and drug treatments

The parental K562 BCR::ABL1+ cell line (K562S) was maintained in RPMI 1640 medium (Lonza) supplemented with 10% fetal calf serum (FCS, Gibco), 1% L-Glutamine and antibiotics in 5% CO_2_ and fully humidified atmosphere at 37°C. K562R cells were generated by progressively increasing imatinib concentration (from 0.05 μM to 10 μM) in the culture medium. K562R cells were then maintained using a constant concentration of 10 μM of imatinib in the medium. Drug resistance was confirmed by clonogenic assays and BCR::ABL1 dephosphorylation (indicating that survival in the presence of imatinib was sustained by a BCR::ABL1-independent mechanism of TKI resistance) was confirmed by Western blotting (16).

Samples from 4 CML patients in BC showing resistance to multiple lines of TKI therapy were also included in our study. All patients were characterized by having no BCR::ABL1kinase domain mutations detectable either pre-therapy or after the first or the second line of therapy (**Table S1**). Samples were collected after written informed consent. This study was conducted according to the principles of the Declaration of Helsinki and was approved by the Independent Ethics Committee of the “S. Orsola-Malpighi” University Hospital of Bologna (protocol 112/2014/U/Tess). The mononuclear cell fraction (MCF) was isolated from bone marrow or peripheral blood samples of patients and peripheral blood apheresis products of 8 healthy donors (HD) by Ficoll-Hypaque density gradient centrifugation. CD34+ cells were isolated by immunomagnetic separation (mini-MACS, Miltenyi Biotec). Briefly, the MCF was incubated at 4° C for 30’ with magnetic microbeads coated with an anti-CD34 antibody (Miltenyi Biotec). CD34+ cells were flown through a separation column in a magnetic field. Cell purity was assessed using flow cytometry with an anti-CD34-FITC antibody (BD Biosciences) and was found to be >84% in all cases (data not shown).

Imatinib (1 μM), danusertib (1 μM) and volasertib (1 μM) were used to inhibit BCR::ABL1, Aurora kinase A and PLK1, respectively. The Wee1 inhibitor AZD1775 (1 μM) was used alone or in combination with danusertib (500 nM) and volasertib (500 nM).

The drug concentrations used for cell cycle analysis, apoptotic assays, clonogenic assays, Western blotting experiments and IF were determined by performing growth curves with increasing doses of each drug (from 200 nM to 1 μM) and different exposure times (from 24h to 72h) (data not shown). The optimal times and concentrations subsequently selected for the Western blotting experiments were those that allowed to obtain a cell death rate ranging between 30 and 60%, so that the effects of death could be studied minimizing the degradation of cell proteins by endonucleases.

### Analysis of cell cycle distribution and apoptosis

Cell cycle distribution analysis was performed on 5 × 10^5^ cells fixed overnight in 70% ethanol and treated with 1 μg/μL propidium iodide (PI) and RNAse (both from Sigma) at 37°C for 30 min. Apoptotic cells were recognized by cytofluorimetric analysis of fluorescinated Annexin V and PI uptake (Roche). Cell fluorescence and PI uptake were measured by mean of a FACScan flow cytometer (set at 488 nm excitation and 530 nm bandpass filter wavelength for fluorescin detection or >580 nm for PI detection) and two different dedicated softwares were used to analyze results (Modfit and Diva software from Beckton Dickinson) (29). Data obtained from cytotoxicity assays were analyzed using the CompuSyn software (ComboSyn, Inc; Paramus, NJ, USA) applied to calculate the efficacy of different drugs alone or in combination in cell lines and in primary patient cells. Combination indexes were calculated using the same software.

### Protein analyses

Whole cell lysates were used for protein analyses (Western blotting) and evaluation of protein post-translational modifications. Briefly, 10x10^6^ cells before and after treatments, were resuspended in 200µl of lysis solution (20 mM Tris-HCl, pH 7.5) and maintained in constant agitation for 30 min at 4°C. Lysates were then centrifuged in a microcentrifuge at 4°C for 30 min at 12,000 rpm. The supernatant was collected and placed in a fresh tube kept on ice, and the pellet discarded. 40µg of the whole cell lysates were resolved by SDS PAGE. Gels were removed from the electrophoresis apparatus and transferred onto a PVDF transfer membrane. Signal intensities in single blots obtained in three separate experiments were revealed by means of the ChemiDoc XRS+ Gel Imaging System (BioRad) equipped with a dedicated software (Image Lab, BioRad).

### Clonogenic assays

Drug cytotoxicity was evaluated in cell lines (K562S and K562R) and CD34+ progenitor cells from BC patients or a pool of 8 healthy donors (HD) by clonogenic assays. The reduction of colony number (generated in 0.9% methylcellulose supplemented with 30% fetal calf serum) in the presence of increasing doses of imatinib (0.025-0.1 µM), danusertib (0.025-0.1 µM) and volasertib (0.025-0.1 µM) was assessed after 14 days of incubation at 37°C in a fully humidified atmosphere and 5% CO_2_. Colonies were counted and the number of colonies obtained in the control sample was conventionally set at 100. The ratio between the number of colonies counted in the different treatment conditions and the number of colonies counted in the control sample was used to build survival curves. Nonlinear regression analyses (GraphPad Prism; GraphPad Software Inc.) were used to calculate the lethal doses (LD50) of the different drugs and combinations.

### IF analysis

Cells set on poly-L-lysine-coated glass slides were fixed with 3% paraformaldehyde for 10’ at 37°C, washed with 0.1M glycine in phosphate-buffered saline (PBS), permeabilized in 70% ice-cold ethanol for 2’ at -20° C and incubated overnight at 4°C with primary anti-ɤH2AX and anti-RAD51 antibodies, or with anti-β-tubulin Alexa Fluor 555 Conjugate which allows to highlight the formation of the mitotic spindle and to calculate the mitotic index. Slides were then incubated with a secondary anti-rabbit antibody conjugated with Alexa Fluor 568 for 2 h at room temperature, and a subsequent anti-mouse fluoresceinated 6-diamidino-2-phenylindole (DAPI) antibody was used to stain the nuclear compartment. IF analyses were performed using an Axiovert 40 CFL microscope (Zeiss). Images were acquired with a 100X objective and analyzed with the AxioVision software. The mitotic index was calculated as the ratio between the number of cells undergoing mitosis and the number of cells not undergoing mitosis.

### Statistics

Data are presented as mean ± standard deviation (SD) and were analyzed for statistical significance by Student t-test (GraphPad Prism Software). A P value <0.05 was considered as statistically significant.

## Results

### Inhibition of Aurora kinase A or PLK1 arrests cell proliferation and favors apoptosis in both TKI-sensitive and -resistant CML cells

**Figure 1:**
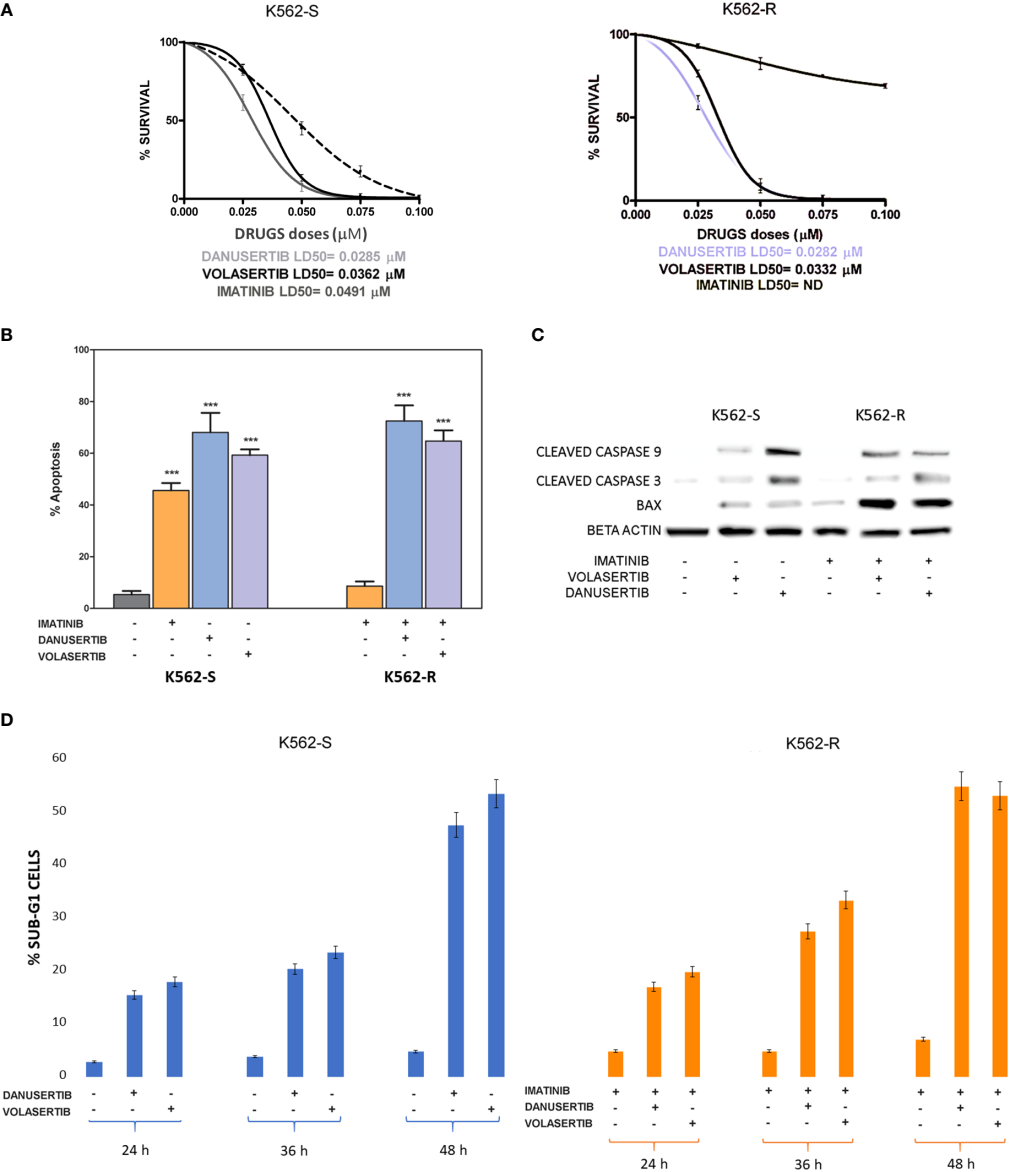
Effects of Aurora kinase A and PLK1 inhibition on CML cell proliferation. **(A)** Dose-dependent inhibition of K562S (left panel) and K562R (right panel) cells in 14-days methylcellulose colony-forming assays. Cells were treated with increasing concentrations of imatinib, danusertib (Aurora kinase A inhibitor) and volasertib (PLK1 inhibitor). Danusertib treatment is represented by the grey line, volasertib is represented by the dark line and imatinib by the dotted line **(B)** Flow cytometry analysis of Annexin-V/PI positive cells. K562S and K562R cells were treated with imatinib (1 μM), danusertib (1 μM) and volasertib (1 μM) for 24 hours. **(C)** Western blotting analysis of apoptosis-related proteins in K562S and K562R cells treated with 1 μM of imatinib, danusertib and volasertib for 24 hours. An antibody against β-actin was used as loading control. Densitometric analyses of each gel were performed by using a dedicated software (ImageJ). Results are detailed in the Supplementary section. **(D)** Flow cytometry analysis of sub-G1 cells. K562S and K562R cells were treated with 1 μM of danusertib or volasertib for 24, 36, 48 hours. Results of clonogenic assays on K562S and K562R represent the mean of three biological replicates. Western blotting assays were performed starting from lysates obtained by three independent drug treatments for each sample. ***p< 0.001.

We have already demonstrated that the serine/threonine kinase PLK1 is hyper-activated in the progenitor cell compartment of CML consisting of CD34+/BCR::ABL1+ cells, and that together with Aurora kinase A up-modulation, it contributes to TKI resistance (15). To better understand the role of PLK1 and Aurora kinase A in sustaining proliferation of CML cells, we performed clonogenic assays in imatinib-sensitive (K562S) and imatinib-resistant (K562R) K562 cells in the presence of danusertib or volasertib (that inhibit Aurora kinase A and PLK1, respectively). While imatinib spared K562R cells even at the dose of 8 µM, both Aurora kinase A and PLK1 inhibition induced a dramatic dose-dependent reduction in colony formation, with LD50 ranging from 0.0282 to 0.0362 µM in both K562S and K562R cells (**Figure 1A**, raw data were reported in **Supplementary material Tables S2, S3**). In agreement with our previous results, inhibition of cell proliferation was associated with increased apoptosis and caspase activation (**Figures 1B, C**). Interestingly, the greatest upregulation of the apoptotic activator BAX was observed in K562R cells following danusertib and volasertib treatments, indicating that both Aurora kinase A and PLK1 play a key role in sustaining resistant CML cells, since their inhibition is a stressful condition that triggers apoptosis. Finally, a time kinetic profiling of cell cycle after 6- and 12-hour treatment with danusertib and volasertib did not show any difference in cell cycle distribution, while after 36 and 48 hours of treatment the sub-G1 peak was too high to appreciate any difference in cell cycle progression (**Figure 1D**).

### Aurora kinase A or PLK1 inhibition induces cell cycle arrest and affects G2/M checkpoint proteins

**Figure 2:**
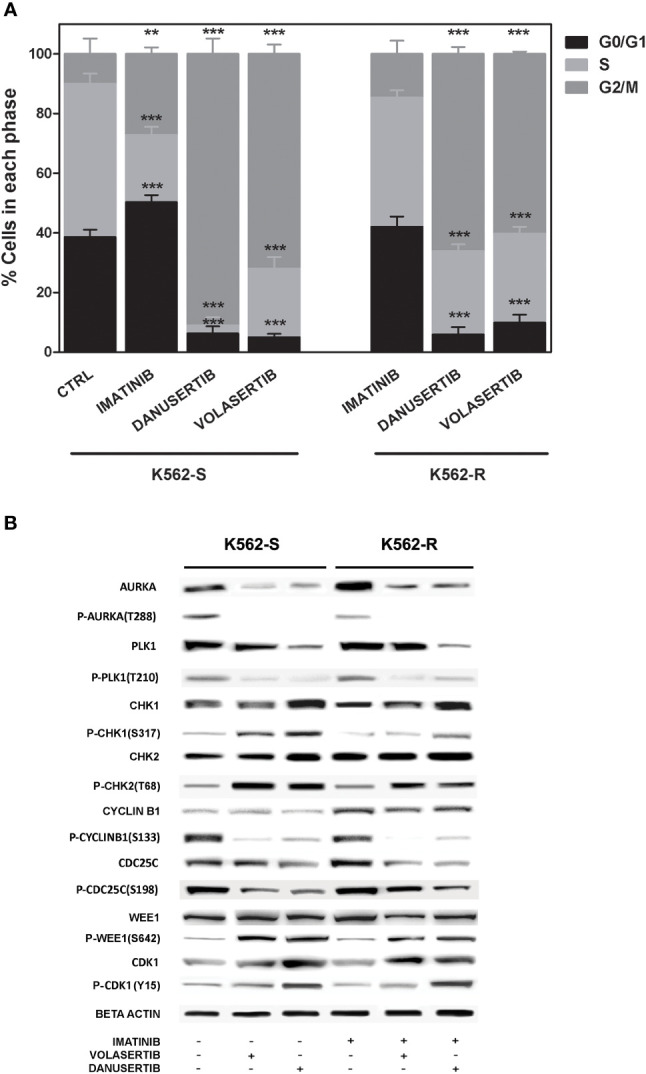
Effects of Aurora kinase A and PLK1 inhibition on cell cycle. **(A)** Flow cytometry analysis of cell cycle. K562S and K562R cells were treated with 1 µM of imatinib, danusertib or volasertib for 24 hours. Asterisks indicate a significant difference between control (for K562S) and imatinib-treated (for K562R) cells and between treatments with danusertib and volasertib. **(B)** Western blotting analysis of cell cycle-related proteins in K562S and K562R cells treated with 1 µM of imatinib, danusertib and volasertib for 24 hours. Beta-Actin was used as loading control. Densitometric analyses of each gel were performed by using a dedicated software (ImageJ). Results are detailed in the Supplementary section. All Western blotting assays were performed starting from lysates obtained by three independent drug treatments for each sample.. **p< 0.01, ***p< 0.001, p ≤ 0.05.

To further investigate the role of Aurora kinase A and PLK1 in CML cell proliferation, we next examined the impact of their inhibition on cell cycle. Both danusertib and volasertib treatment dramatically increased G2/M phase cells regardless of imatinib sensitivity (**Figure 2A**). Due to the role of Aurora kinase A and PLK1 in driving cell-cycle progression, in normal cells their expression and activity is tightly controlled, peaking during G2 and mitosis and dropping at mitotic exit (30). We have previously reported that both Aurora kinase A and PLK1 are up-modulated and hyper-activated in imatinib-resistant BCR::ABL1 positive CML cells (15). To confirm the efficacy of danusertib and volasertib in Aurora kinase A and PLK1 inhibition and to assess if the molecular effects of drug administration are on-target, we performed Western blotting assays testing Aurora kinase A and PLK1 expression and phosphorylation (**Figure 2B**). Results demonstrated that danusertib and volasertib indeed induce down-modulation and de-phosphorylation of Aurora kinase A and PLK1, respectively. To determine the functional consequences of Aurora kinase A and PLK1 inhibition, we used Western blotting to assess the phosphorylation of several proteins involved in cell cycle control including CHK-1, CHK-2, cyclin B1, WEE1 and CDK1 (**Figure 2B**). We observed increased levels of active phospho (p)-CHK1/2 proteins, that are required for checkpoint-mediated cell cycle arrest to ensure the integrity of the genome before DNA replication, and of nuclear p-cyclin B1, that regulates G2/M transition. As expected, p-cyclin B1 on S133 and p-CDC25C on S198 were reduced after PLK1 inhibition. These observations confirm the on-target effects of volasertib 1 µM for 24 h. Moreover, active p-WEE1, that regulates CDK1 activity, was found to be increased, thus preventing the progression into mitosis. Collectively, these data support the hypothesis that Aurora kinase A and PLK1 over-expression and hyper-activation induce DNA damage causing G2/M cell cycle arrest. A time kinetic profiling of cell cycle after Aurora inhibition (24, 36, 48 hours) showed that danusertib significantly induced polyploidy in all surviving cells, thus ultimately causing apoptotic cell death (**Figure 1D**).

**Figure 3:**
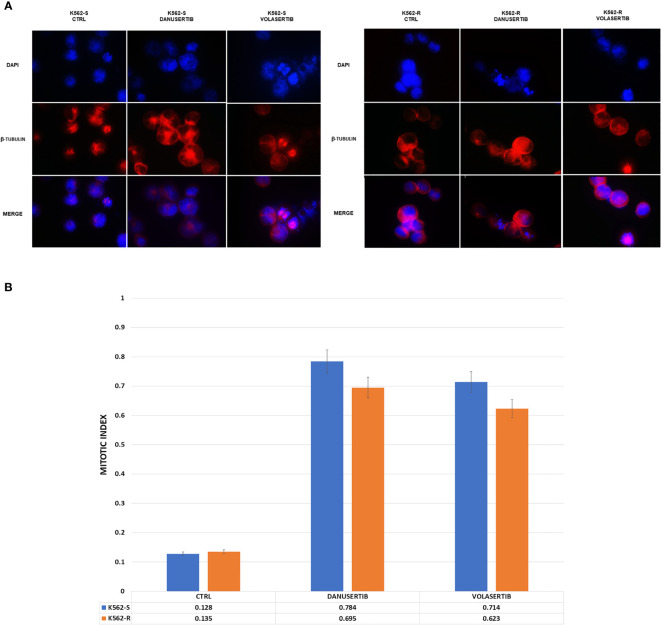
Effects of Aurora kinase A and PLK1 inhibition on cell division. **(A)** Immunofluorescence analysis of mitotic cells after staining with DAPI (4’,6-Diamidino-2-Phenylindole) and β-tubulin in K562S and K562R cells treated with 1 μM danusertib or volasertib for 24 hours. K562R cells were maintained under imatinib (10 μM) pharmacological pressure. The mitotic index was calculated as the ratio of the number of mitotic cells/number of total cells. **(B)** A bar graph representing mitotic index values of K562S (light blue bars) and K562R (orange bars) cell lines. Results are the mean of three independent experiments.

To distinguish mitotic cells from those in G2 phase and to determine whether the cell cycle block is actually in G2 phase or in early mitosis, we labeled K562S and K562R cell lines before and after treatment with danusertib and volasertib 1 µM for 24 hours with an anti-β-tubulin (a component of mitotic spindle) antibody and DAPI. Both danusertib and volasertib induced a significant accumulation of K562S and K562R cells in mitotic phase (**Figure 3**).

### WEE1 inhibition following Aurora kinase A or PLK1 inhibition dramatically increases DNA damage

**Figure 4:**
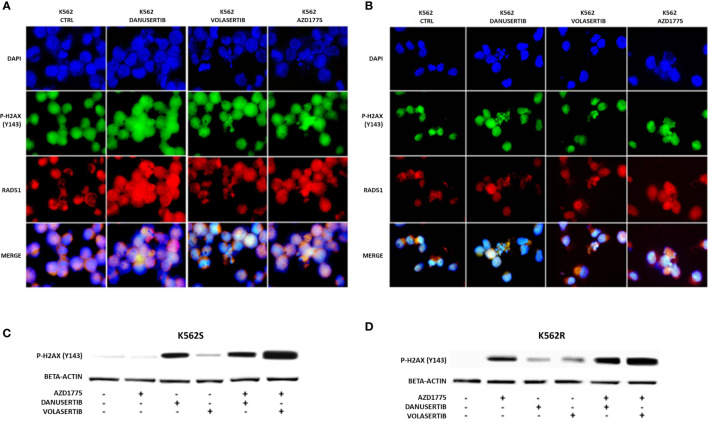
Effects of Aurora kinase A, PLK1 and WEE1 inhibition on DNA damage. Panels **(A, B)** show immunofluorescence analysis of phosphorylated histone 2A.X (ɤH2AX, green) and Rad51 (red) in K562S **(A)** compared to K562R **(B)** after 24 hours of treatment with danusertib, volasertib and AZD1775. The K562R cell line is cultured maintaining a constant imatinib concentration in the medium. Staining with DAPI (4’,6-Diamidino-2-Phenylindole) indicates the nuclear localization of ɤH2AX and RAD51. Panels **(C, D)** show Western blotting analysis of phosphorylated histone 2A.X revealing the effects on DNA damaged cells of danusertib or volasertib alone as compared to 24h priming with danusertib or volasertib alone + 24h combination of danusertib or volasertib+AZD1775. K562R cells were maintained in RPMI additioned with 10 µM imatinib. Beta-actin was used as loading control. Western blotting assays were performed starting from lysates obtained from three independent drug treatments for each sample. Densitometric analyses of each gel were performed by using a dedicated software (ImageJ). Data obtained, are shown in the supplementary file named:”WB densitometric readings”.

Given that Aurora kinase A and PLK1 inhibition induced p-WEE1 up-modulation, we hypothesized that blocking WEE1 could promote mitosis and trigger apoptosis as a result of DNA damage propagation. First, we assessed the DNA damage induced by Aurora kinase A, PLK1 and WEE1 inhibition following treatment with danusertib, volasertib or AZD1775, respectively, as single agents. As detected by IF, in both K562S and K562R cell lines danusertib and volasertib treatments perturbed the mitotic process and induced ɤ-H2AX and Rad51 nuclear foci formation (**Figure 4A**), indicating the presence of DNA double strand breaks and repair activity, respectively. Next, we evaluated a schedule whereby 24h treatment with danusertib or volasertib was followed by 24h combination with AZD1775 to force K562S and K562R cell entry in mitosis. This approach dramatically increased the DNA double strand break marker ɤ-H2AX (**Figure 4B**). These data indicate that WEE1 blockage following Aurora kinase A or PLK1 inhibition propagates genomic instability in both imatinib-sensitive and -resistant cells through successive replication cycles.

### Combined WEE1 and Aurora kinase A or PLK1 inhibition exerts additive effects and dramatically affects LSC viability

**Figure 5:**
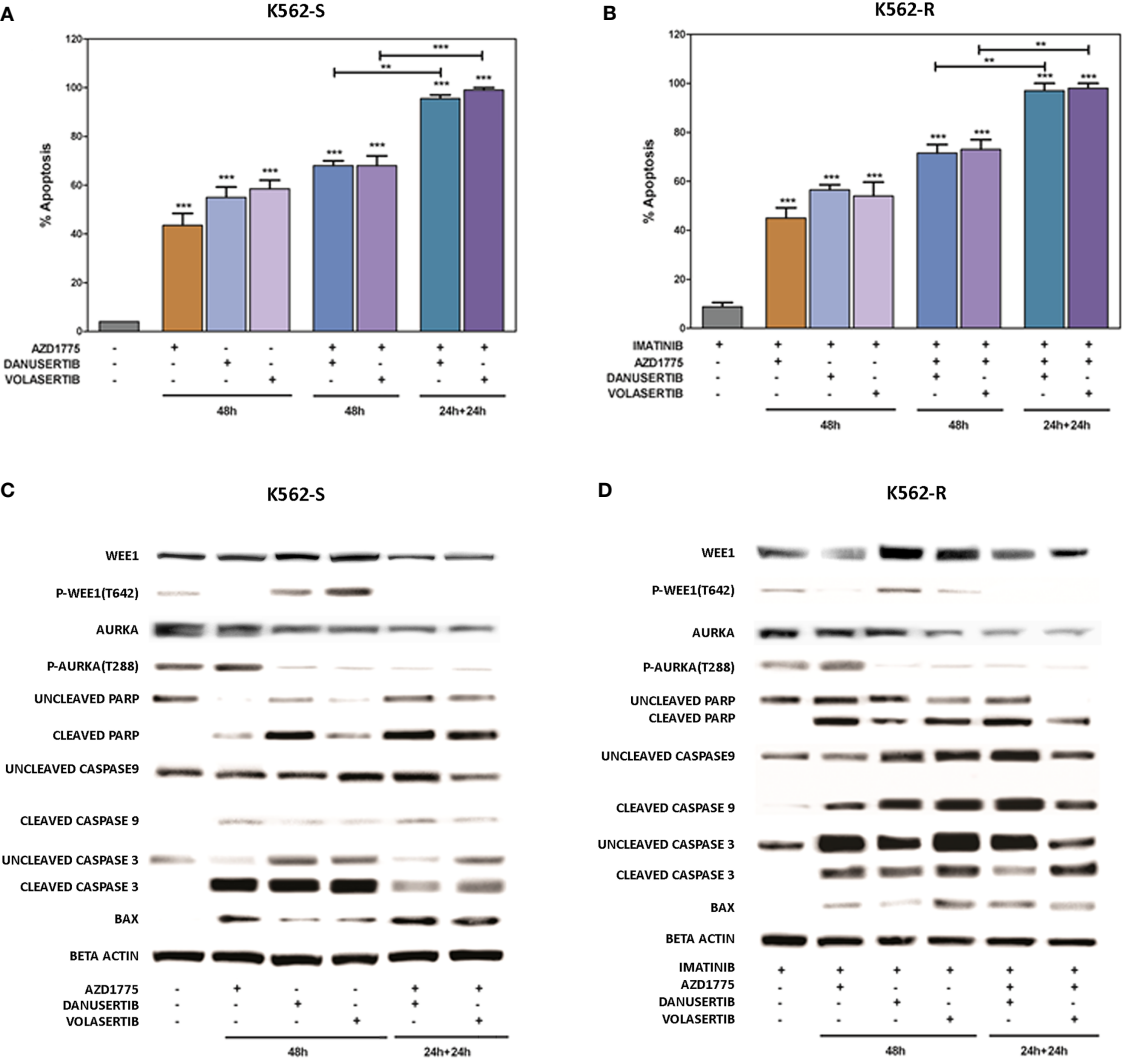
Effects of Aurora kinase A or PLK1 inhibition in combination with Wee1 inhibition. Flow cytometry analysis of apoptosis induction in K562S **(A)** and K562R **(B)** cells following 48h-co-treatment with danusertib or volasertib+AZD1775 (schedule a), or 24h-treatment with danusertib or volasertib followed by 24h-treatment with danusertib or volasertib+AZD1775 (schedule b). Western blot analysis of apoptosis-related proteins in K562S **(C)** and K562R **(D)** cells following treatment with Aurora kinase A, Plk1 or Wee1 inhibitors as single agents or in combination according to schedule a or (b) Beta-actin was used as loading control. Apoptotic cell death evaluations were represented as a mean of three biological replicates. Similarly, all Western blotting assays were performed starting from lysates obtained by three independent drug treatments for each sample. Densitometric analyses of each gels were performed by using a dedicated software (ImageJ). Results are detailed in the Supplementary section. **p< 0.01, ***p< 0.001, p ≤ 0.05.

To further investigate the impact of the accumulation of damaged DNA, we used flow cytometry to verify induction of apoptosis. We examined and compared the effects of two different schedules: a) 48h-co-treatment with either danusertib or volasertib+AZD1775, and b) 24 h-treatment with danusertib or volasertib followed by 24 h-treatment with danusertib or volasertib+AZD1775. As illustrated in **Figures 5A, B**, while the first schedule induced only a modest increase in apoptotic cells in both K562S and K562R cells, the second one induced a dramatic induction of caspase-dependent apoptosis (**Figures 5 A–D**), indicating that in K562S and K562R cells the apoptotic response is triggered to avoid the propagation of mutations. After cell line treatment with danusertib or volasertib and AZD1775 for 24 + 24 hours with increasing doses of the drugs (0.20-1.0 µM), the combination index (CI; calculated by Compusyn) showed that the combination of danusertib and AZD1775 as well as the combination of volasertib and AZD1775 had additive effects both in K562S and in K562R (CI=1) (**Supplemantary Material: Tables S9–S22**).

**Figure 6:**
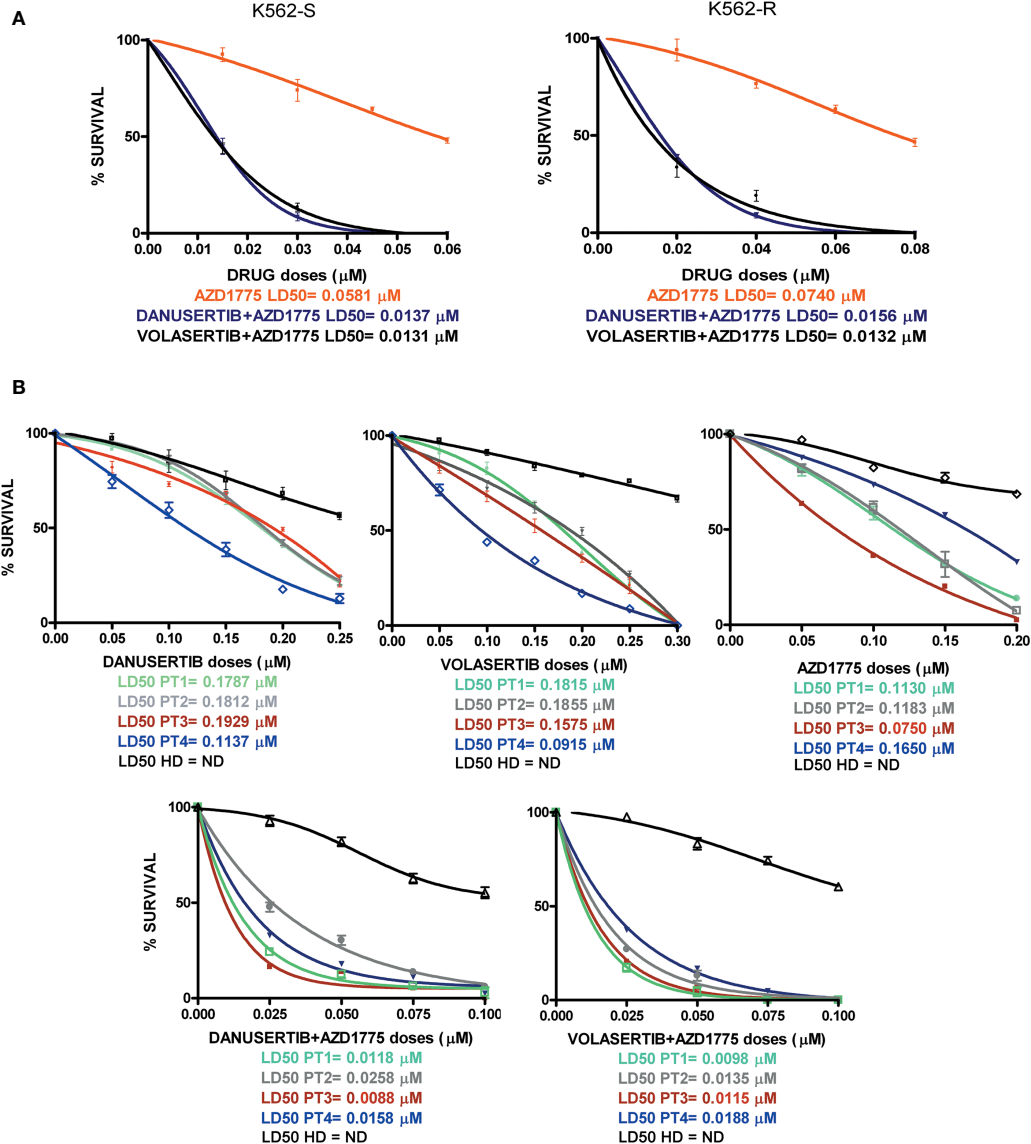
Effects of Aurora kinase A, PLK1 and WEE1 inhibition in K562S and K562R cell lines and in CD34+ cells derived from 4 BC CML patients and a pool of 8 healthy donors (HDs). Dose-dependent inhibition of survival was assessed in 14-day methylcellulose colony-forming assays. **(A)** K562S and -R cells and **(B)** CD34+ cells were treated with increasing concentrations of danusertib (Aurora kinase A inhibitor), volasertib (PLK1 inhibitor), AZD1775 (WEE1 inhibitor) danusertib+AZD1775, volasertib+AZD1775. The green line corresponds to patient 1, the gray line corresponds to patient 2, the red line corresponds to patient 3, the blue line corresponds to patient 4 and the black line corresponds to the pool of 8 healthy donors.

To ascertain whether the combination of danusertib or volasertib and AZD1775 could be an useful therapeutic strategy for BC CML patients, we assessed the effects of Aurora kinase A, PLK1 and WEE1 inhibition on the clonogenic potential of CD34+/BCR::ABL1+ cells isolated from 4 multi-TKI-resistant BC CML patients. Indeed, this cellular fraction is enriched in LSCs, that are intrinsically resistant to TKIs and are supposed to be a source of relapse (10). We found that the combinations significantly reduced the proliferation of CD34+/BCR::ABL1+ primary blasts in all patients. Importantly, neither the single drugs nor the combined treatments significantly impacted on the viability of cells derived from a pool of 8 healthy donors, tested as control (**Figure 6**, raw data were reported in **Supplementary Material Tables S4–S8**). Collectively, our findings suggest that combined administration of Aurora kinase A or PLK1 and WEE1 inhibitors could be a promising strategy to enhance apoptosis in TKI-resistant CML cells.

## Discussion

CML is a hematologic neoplasm sustained by the constitutive activation of the BCR::ABL1 fusion kinase that can successfully be targeted with TKIs, so that most CML patients nowadays obtain durable remissions. However, tumor escape mechanisms may compromise TKI efficacy. The most extensively investigated mechanism is the selection of point mutations in the BCR::ABL1 kinase domain, that turn on BCR::ABL1 TKI activity again by impairing inhibitor binding (31, 32). To counteract this mechanism, two generations of more potent and/or selective ATP-competitive TKIs have been developed over the years, and switching from one TKI to another may rescue response in patients harboring mutations (33). A novel and very selective allosteric BCR::ABL1 inhibitor (asciminib) is also being incorporated in the CML therapeutic armamentarium. However, resistance is frequently driven by BCR::ABL1-independent mechanisms that sustain the proliferation and survival of CML cells regardless of BCR::ABL1 inhibition, finally resulting in disease relapse and eventually progression (1–3). Much less is known about these mechanisms. Therefore, it is extremely important to dissect the cellular signals implicated in BCR::ABL1-independent drug resistance in order to identify novel therapeutic paradigms to treat TKI-resistant patients. This is especially true for the setting of patients who progress from CP to BC, in whom targeting BCR::ABL1 alone is not sufficient anymore and prognosis has remained poor even in the TKI era (34). Moreover, it has been extensively demonstrated that CML LSCs are not dependent on BCR::ABL1 for their survival (35, 36). This highlights the need to investigate the molecular mechanisms alternative to BCR::ABL1 that are responsible for LSCs persistence, in order to make them pharmacologically targetable. Despite intensive research efforts, no novel safe and effective therapeutic strategies have been clinically explored yet.

In this study, we built on our previous findings showing that both Aurora kinase A and PLK1 are hyper-activated in an imatinib-resistant CML K562 (K562R) cell line (16). In particular, we had demonstrated that our imatinib-resistant cell line was a model of BCR::ABL1-independent drug resistance, since neither BCR::ABL1 itself nor its downstream substate Crk-L were phosphorylated in K562R cells maintained in imatinib 10 µM – which indicated effective BCR::ABL1 inhibition by imatinib. Moreover, we had very interestingly shown that Aurora kinase A and PLK1 over-expression and hyper-activation is also a feature of the CD34+ compartment, where LSCs are known to reside (16). Additionally, we had found that PLK1 activates FoxM1, a proliferation-associated transcription factor involved in hematopoietic stem cell (HSC) self-renewal (16). Whenever novel treatment paradigms are to be identified in relatively rare patient populations, the most straightforward approach is the repurposing of drugs in advanced clinical development for other conditions. Starting from our preliminary data and from previous studies (37, 38), we reasoned that this might indeed be the case for Aurora kinase A and PLK1 inhibitors. We thus decided to explore the anti-proliferative effects of danusertib and volasertib, two selective inhibitors of Aurora kinase A and PLK1, respectively. We found that in both TKI-sensitive and -resistant CML cell lines, these two inhibitors were able to impair proliferation and induce a significant degree of caspase-dependent apoptosis. Interestingly, as compared to imatinib, these two drugs act by exploiting alternative pathways. Indeed, both danusertib and volasertib induced a dramatic increase of cells in G2/M phase. This observation prompted us to test, as a subsequent step, different combinations strategies of danusertib or volasertib and a WEE1 inhibitor (AZD1775) with the aim to increase the percentage of apoptotic cell death and to minimize cell escape mechanisms and eventually drug resistance. WEE1 is a protein kinase that is over-expressed and hyper-activated in G2 phase. We thus decided to use a WEE1 inhibitor at the exact timepoint in the cell cycle when WEE1 is hyper-activated: after 24 hours of inhibition of Aurora kinase A or PLK1. The combination of drugs we designed was indeed found to be very effective if compared to the administration of the single drugs.

We detected a significant modulation in post-translational modifications of several proteins involved in G2/M transition and regulated by Aurora kinase A or PLK1, such as cyclin B1 (phosphorylated on ser133) and CDC25C. Notably, this was also observed in our cell line model recapitulating BCR::ABL1-independent imatinib resistance. All the effects observed in the present study are attributable solely to Aurora kinase A or PLK1 inhibition, suggesting that danusertib or volasertib could be an alternative strategy to treat relapsed/resistant CML patients. Importantly, the combination of danusertib or volasertib + AZD1775 was effective not only in CML cell lines, but also in CD34+/BCR::ABL1+ cells (representative of the LSC compartment) isolated from 4 BC CML patients. We ruled out any effect of the combinations on CD34+ progenitors obtained from a pool of healthy donors. This suggests that Aurora kinase A and PLK1 drive survival signals capable to induce persistence of LSCs but not necessary of HSCs.

Our mechanistic studies explain why Aurora kinase A and PLK1 inhibition alone or in combination with WEE1 inhibition induce apoptosis in TKI-sensitive and -resistant BCR::ABL1+ cell lines and in multi-TKI-resistant BC CML patient cells. We propose a model that describes the roles of Aurora kinase A and PLK1 over-expression and hyper-activation in signaling pathways that operate in CML cells and the effects of their inhibition. In this model, our findings suggest that both Aurora kinase A and PLK1 have important roles in activating proliferation and uncontrolled cell division and in inducing genomic instability. Based on our findings, we propose that combined targeting of Aurora kinase A or PLK1+WEE1 may be an excellent strategy for inducing apoptosis in CML cells whose resistance is driven by BCR::ABL1-independent mechanisms, or where all TKIs have failed, including advanced stages of CML.

Interestingly, danusertib has also a documented potent activity against ABL1 and BCR::ABL1, including the T315I mutant: IC50 values in BaF3 p210 wild-type and BaF3 p210 T315I cells have been reported to be 0.36 and 0.12 μM, respectively (39). Danusertib had indeed shown good tolerability and satisfactory clinical activity in phase 1 and 2 studies in advanced CML cases. Therefore, the danusertib+AZD1775 schedule herein proposed would simultaneously inhibit Aurora A, BCR::ABL1 and WEE1, with the dual advantage of counteracting the growth of clones sustained by both BCR::ABL1-dependent and -independent mechanisms of resistance. Thus, danusertib or volasertib associated with AZD1775 may have the potential to effectively address some remaining key unmet needs in CML treatment.

Overall, our results demonstrate at a preclinical level that the combination of danusertib or volasertib and AZD1775 (or other, equivalent inhibitors that could rapidly be repurposed) is a promising option capable to counteract BCR::ABL1-independent TKI resistance and to kill CD34+ LSC progenitors of BC patients, while leaving normal HSCs unaffected. These combinations would thus deserve further investigation at the clinical level in an attempt to improve the poor outcome of multi-TKI-resistant and BC CML patients.

## Data availability statement

The original contributions presented in the study are included in the article/**Supplementary Material**. Further inquiries can be directed to the corresponding author.

## Ethics statement

The studies involving human participants were reviewed and approved by Independent Ethics Committee of the “S. Orsola-Malpighi” University Hospital of Bologna. The patients/participants provided their written informed consent to participate in this study.

## Author contributions

Conceptualization, MM and SS. Data curation, MM and SS. Formal analysis, MM and AL. Funding acquisition, MM, MC, GG and SS. Investigation, MM, SS and CM. Methodology, SS, CM and SB. Project administration, MM, MC, GG and SS. Resources, MM, ED, BS, GG and SS. Software, MM and AL. Supervision, FC, MC and SS. Validation, SS, CM and SB. Visualization, CM, FC, SB, ED, BS and GR. Writing – original draft, MM and AL. Writing – review & editing, FC, MC, GG and SS. All authors have read and agreed to the published version of the manuscript.

## Funding

This study was supported by AIRC IG 2019 (project code 23001) (SS), by the Italian Ministry of Health, “Bando Ricerca Finalizzata 2016” (project GR-2016-02364880) (GG), and by AIL Bologna ODV.

## Conflict of interest

The authors declare that the research was conducted in the absence of any commercial or financial relationships that could be construed as a potential conflict of interest.

## Publisher’s note

All claims expressed in this article are solely those of the authors and do not necessarily represent those of their affiliated organizations, or those of the publisher, the editors and the reviewers. Any product that may be evaluated in this article, or claim that may be made by its manufacturer, is not guaranteed or endorsed by the publisher.
