# Supplementary material for: Polo-like kinase-1, Aurora kinase A and WEE1 kinase are promising druggable targets in CML cells displaying BCR::ABL1-independent resistance to tyrosine kinase inhibitors
Source: Front Oncol. 2022 Aug 5;12:901132. doi: 10.3389/fonc.2022.901132 (PMC9391055; doi:10.3389/fonc.2022.901132)
Supplement: Supplementary file 1 [file DataSheet_1.pdf]

frontiers

SUPPLEMENTARY MATERIAL

Supplementary Material and Methods

Supplementary Table S1 - Characteristicts of the 4 BC patients included in the study.

CASE	AGE	GENDER	STAGE OF DISEASE	BCR::ABL1 MUTATION	TREATMENT
PT1	65	F	CML-LY-BC	ND	IM, NIL
PT2	51	M	CML-LY-BC	ND	IM, NIL
PT3	71	M	CML MY-BC	ND	NIL, DAS
PT4	74	M	CML MY-BC	ND	IM, DAS

Abbreviations: PT: patient, F: female, M: male, CML: chronic myeloid leukemia, LY-BC: lymphoid blast crisis, MY-BC: myeloid blast crisis, ND: not detected by Sanger sequencing, IM: imatinib, NIL: nilotinib, DAS: dasatinib.

Supplementary Results

Clonogenic assays

Clonogenic assays were carried out using 5000 cells for each drug condition in K562S and K562R cell lines, and using 10000 cells for each drug condition in CD34+ progenitors. Colonies formed after 14 days of culture in methylcellulose semisolid medium were counted and, in order to obtain survival curves easier to read, the number of colonies counted in the control sample was conventionally set at 100. The ratio between the number of colonies counted in the different treatment conditions and the number of colonies counted in the control sample was used to build the survival curves shown in Figures 1 and 6. The raw data are detailed in the Tables blow.

Supplementary Table S2: Dose-dependent inhibition effects of treatment with AZD1775 alone or in combination with danusertib and volasertib in K562S cells.

K562-S	AZD1775		DANUSERTIB+AZD1775		VOLASERTIB+AZD1775	
0	100	100	100	100	100	100
0.015	90	95	48	42	46	42
0.03	70	78	10	7	15	12
0.045	63	65	0	0	0	0
0.06	47	49	0	0	0	0

Supplementary Table S3: Dose-dependent inhibition effects of treatment with imatinib, danusertib and volasertib in K562R cells

K562-R	IMATINIB		DANUSERTIB		VOLASERTIB	
0	100	100	100	100	100	100
0.025	94	92	62	56	75	78
0.05	80	85	12	6	10	7
0.075	75	75	0	0	3	1
0.1	68	70	0	0	0	0

Supplementary Table S4: Dose-dependent inhibition effects of treatment with AZD1775 in CD34+ progenitor cells from BC patients or a pool of 8 healthy donors (HDs).

AZD 1775	HDs		PT1		PT2		PT3		PT4	
0	100	100	100	100	100	100	100	100	100	100
0.05	95.6	98.5	90	85.4	78.1	84.2	80	84.3	62	65
0.1	84.2	80.7	78.1	68.7	62.1	55.1	58.1	64.8	30.4	42.2
0.15	74.5	79.8	55.2	60.1	32.1	30.8	38.4	25.1	15.2	25.1
0.2	69.4	67.8	35.8	30.1	15.8	12	5.8	9.1	0	5

Supplementary Table S5: Dose-dependent inhibition effects of treatment with danusertib in CD34+ progenitor cells from BC patients or a pool of 8 healthy donors (HDs).

DANUSERTIB	HDs		PT1		PT2		PT3		PT4	
0	100	100	100	100	100	100	100	100	100	100
0.05	99.1	95.3	91	92	93.8	94.8	80	84.3	71	78
0.1	89.5	81.2	82	85	84.5	87.6	72.3	74.1	55.4	63.5
0.15	78.6	71.7	66	63	68	64.9	66.7	68.9	35	42.3
0.2	70.5	65.9	40	42	41.2	43.3	50.1	48.7	15.8	19.5
0.25	55	57.7	23	20	23.7	20.6	24.3	19.8	10.3	15.2

Supplementary Table S6: Dose-dependent inhibition effects of treatment with volasertib in CD34+ progenitor cells from BC patients or a pool of 8 healthy donors (HDs).

**VOLASERTIB**	**HDs**		**PT1**		**PT2**		**PT3**		**PT4**	
0	100	100	100	100	100	100	100	100	100	100
0.05	98.2	97.1	93.8	87.6	84	82	81	85	74.3	68.4
0.1	89.9	92.2	85	80.4	75	69	66	70	45.6	42.1
0.15	85.4	82.3	65	61.8	64	60	50	55	35.2	33.1
0.2	79.4	78.7	40	36.1	51	48	34	37	15.2	18.6
0.25	75.5	76.4	23	20.6	25	28	18	24	10.5	7.1
0.3	68.2	65.6	0	0	0	0	0	0	0	0

Supplementary Table S7: Dose-dependent inhibition effects of combined treatment with danusertib and AZD1775 in CD34+ progenitor cells from BC patients or a pool of 8 healthy donors (HDs).

**DAN+AZD**	HDS		PT1		PT2		PT3		PT4	
0	100	100	100	100	100	100	100	100	100	100
0.025	95.6	90	35.8	30.1	50.2	45.3	23.1	25.3	17.7	15.2
0.05	84.2	79.5	20.1	15.8	32.8	27.9	10.5	14.6	5.06	8.4
0.075	65.2	60.1	12.5	9.8	12.6	14.9	5.5	7.2	6.3	4.2

0.1	58.1	52.3	5	0	7	5	5.8	0	0	0

Supplementary Table S8: Dose-dependent inhibition effects of combined treatment with volasertib and AZD1775 in CD34+ progenitor cells from BC patients or a pool of 8 healthy donors (HDs).

VOL +AZD	HDS		PT1		PT2		PT3		PT4	
0	100	100	100	100	100	100	100	100	100	100
0.025	98.2	97.1	42.1	32.6	25.8	28.4	19.5	15.1	22.8	18.5
0.05	80.1	86.4	18.4	15.3	10.3	15.8	7	2	5.06	8.4
0.075	72.1	76.4	7.4	2.3	3	0	0	0	0	0
0.1	62.3	58.5	0	0	0	0	0	0	0	0

IC50 and CI values

A dose-escalation experiment was performed to define the drug doses required to induce sub-lethal effects in K562S and K562R cell lines. For this purpose. each drug. alone or in combination. was added at scalar doses into the cell culture medium. starting from 0.2 to 1 μM. After 24+24 hours treatment cells were evaluated for Annexin V uptake and the percentage of residual living cells was used to calculate IC50 values by using a dedicated software (Compusyn). Compusyn reports are shown below.

Supplementary Table S9: Data for Drug: AZD1775 [μM] in K562S cell line

Dose (μM)	Effect (% of living cells)
0.2	0.92
0.5	0.87
0.8	0.69
1.0	0.55

Supplementary Table S10: Data for Drug: volasertib [μM] in K562S cell line

Dose (μM)	Effect (% of living cells)
0.2	0.85
0.5	0.68
0.8	0.51
1.0	0.43

Supplementary Table S11: Data for Drug: danusertib [μM] in K562S cell line

Dose (μM)	Effect (% of living cells)
0.2	0.88
0.5	0.72
0.8	0.53
1.0	0.48

Supplementary Table S12: Data for Drug Combo: AZD1775+danusertib [1:1] in K562S cell line

Dose (μM)	Effect (% of living cells)
0.2	0.62
0.5	0.54
0.8	0.27
1.0	0.13

Supplementary Table S13: CI for Drug Combo: AZD1775+danusertib [1:1] in K562S cell line

Total Dose (μM)	Effect (% of living cells)	CI Value
0.4	0.62	0.65277
1.0	0.54	1.06118
1.6	0.27	0.76950
2.0	0.13	0.58012

Supplementary Table S14: Data for Drug Combo: AZD1775+volasertib [1:1]) in K562S cell line

Dose (μM)	Effect (% of living cells)
0.2	0.60
0.5	0.51
0.8	0.19
1.0	0.10

Supplementary Table S15: CI for Drug Combo: AZD1775+volasertib [1:1]) in K562S cell line

Total Dose (μM)	Effect (% of living cells)	CI Value
0.4	0.60	0.50261
1.0	0.51	1.00001
1.6	0.19	0.65120
2.0	0.10	0.42010

Supplementary Table S16: Data for Drug: AZD1775 [μM] in K562R cell line

Dose (μM)	Effect (% of living cells)
0.25	0.97
0.5	0.91
0.75	0.78
1.0	0.56

Supplementary Table S17: Data for Drug: volasertib [μM] in K562R cell line

Dose (μM)	Effect (% of living cells)
0.2	0.91
0.5	0.72
0.8	0.61

Dose (μM)	Effect (% of living cells)
1.0	0.48

Supplementary Table S18: Data for Drug: danusertib [μM] in K562R cell line

Dose (μM)	Effect (% of living cells)
0.2	0.81
0.5	0.68
0.8	0.47
1.0	0.35

Supplementary Table S19: Data for Drug Combo: AZD1775+danusertib [1:1] in K562R cell line

Dose (μM)	Effect (% of living cells)
0.2	0.57
0.5	0.49
0.8	0.21
1.0	0.09

Supplementary Table S20: CI for Drug Combo: AZD1775+danusertib [1:1] in K562R cell line

Total Dose (μM)	Effect (% of living cells)	CI Value
0.4	0.57	0.61423
1.0	0.49	1.01253
1.6	0.21	0.82475
2.0	0.09	0.52140

Supplementary Table S21: Data for Drug Combo: AZD1775+volasertib [1:1] in K562R cell line

Dose (μM)	Effect (% of living cells)
0.2	0.60
0.5	0.51

Dose (µM)	Effect (% of living cells)
0.8	0.19
1.0	0.10

Supplementary Table S22: CI for Drug Combo: AZD1775+volasertib [1:1] in K562R cell line

Total Dose (µM)	Effect (% of living cells)	CI Value
0.4	0.60	0.50261
1.0	0.51	1.00001
1.6	0.19	0.82456
2.0	0.10	0.42010
