# Supplementary material for: Polo-like kinase-1, Aurora kinase A and WEE1 kinase are promising druggable targets in CML cells displaying BCR::ABL1-independent resistance to tyrosine kinase inhibitors
Source: Front Oncol. 2022 Aug 5;12:901132. doi: 10.3389/fonc.2022.901132 (PMC9391055; doi:10.3389/fonc.2022.901132)
Supplement: Supplementary file 2 [file Presentation_1.pdf]

FIGURE 1

CASP-9

0	K562-S CTRL
7 181 075	K562-S VOLASERTIB
53 949 945	K562-S DANUSERTIB
0	K562-R CTRL
27 472 066	K562-R VOLASERTIB
16 297 167	K562-R DANUSERTIB

CASP 3

1 259 962	K562-S CTRL
6 027 924	K562-S VOLASERTI
43 228 823	K562-S DANUSERTI
446 042	K562-R CTRL
6 490 217	K562-R VOLASERTI
28 659 773	K562-R DANUSERTI

BAX

0	K562-S CTRL
7807267	K562-S VOLASERTIB
6176368	K562-S DANUSERTIB
3 239	K562-R CTRL
59 356 359	K562-R VOLASERTIB
51 780 309	K562-R DANUSERTIB

BETA ACTIN

55 797 116	K562-S CTRL
56 712 894	K562-S VOLASERTI
54 793 045	K562-S DANUSERTI
44 687 066	K562-R CTRL
48 366 258	K562-R VOLASERTI
47 880 773	K562-R DANUSERTI

FIGURE 2

AURORA KINASE A

49 232 664	K562-S CTRL
6 957 317	K562-S VOLASERTIB
11 696 510	K562-S DANUSERTIB
80 732 208	K562-R CTRL
18 484 187	K562-R VOLASERTIB
22 765 279	K562-R DANUSERTIB

CHK1

49 564 087	K562-S CTRL
45 191 087	K562-S VOLASERTI
87 575 037	K562-S DANUSERTI
47 181 246	K562-R CTRL
50 138 936	K562-R VOLASERTI
78 385 551	K562-R DANUSERTI

P-AURORA KINASE A(T288)

66 159 915	K562-S CTRL
0	K562-S VOLASERTIB
0	K562-S DANUSERTIB
26 644 380	K562-R CTRL
0	K562-R VOLASERTIB
0	K562-R DANUSERTIB

P-CHK1(S317)

13 313 681	K562-S CTRL
53 724 501	K562-S VOLASERTI
82 606 016	K562-S DANUSERTI
5 479 903	K562-R CTRL
10 025 752	K562-R VOLASERTI
40 899 723	K562-R DANUSERTI

PLK1

71 757 007	K562-S CTRL
56 880 856	K562-S VOLASERTIB
17 007 167	K562-S DANUSERTIB
76 429 936	K562-R CTRL
57 246 350	K562-R VOLASERTIB
9 887 489	K562-R DANUSERTIB

CHK2

51 675 208	K562-S CTRL
57 183 894	K562-S VOLASERTI
76 141 116	K562-S DANUSERTI
75 007 773	K562-R CTRL
80 480 459	K562-R VOLASERTI
104 124 673	K562-R DANUSERTI

P-PLK1(T210)

74 696 697	K562-S CTRL
11 016 673	K562-S VOLASERTIB
11 102 451	K562-S DANUSERTIB
66 098 886	K562-R CTRL
6 701 401	K562-R VOLASERTIB
18 490 421	K562-R DANUSERTIB

P-CHK2(T68)

16 077 711	K562-S CTRL
96 964 238	K562-S VOLASERTI
78 000 116	K562-S DANUSERTI
24 869 317	K562-R CTRL
59 499 894	K562-R VOLASERTI
45 437 309	K562-R DANUSERTI

FIGURE 2

CYCLIN B1

17 247 430 K562-S CTRL
26 612 522 K562-S VOLASERTIB
14 301 986 K562-S DANUSERTIB
80 694 350 K562-R CTRL
50 748 158 K562-R VOLASERTIB
66 380 329 K562-R DANUSERTIB

CYCLIN B1(S133)

77 235 279 K562-S CTRL
2 650 489 K562-S VOLASERTIB
9 485 823 K562-S DANUSERTIB
55 734 522 K562-R CTRL
102 192 K562-R VOLASERTIB
4 629 368 K562-R DANUSERTIB

CDC25C

50 189 208 K562-S CTRL
44 745 187 K562-S VOLASERTIB
28 368 208 K562-S DANUSERTIB
73 962 037 K562-R CTRL
16 257 702 K562-R VOLASERTIB
10 969 317 K562-R DANUSERTIB

CDC25C(S198)

72 595 622 K562-S CTRL
22 136 016 K562-S VOLASERTIB
21 358 702 K562-S DANUSERTIB
68 257 856 K562-R CTRL
41 129 794 K562-R VOLASERTIB
24 910 066 K562-R DANUSERTIB

WEE1

73 298 108 K562-S CTRL
83 748 454 K562-S VOLASERTIB
64 116 664 K562-S DANUSERTIB
82 323 614 K562-R CTRL
43 123 714 K562-R VOLASERTIB
72 941 128 K562-R DANUSERTIB

P-WEE1(S642)

10 252 974 K562-S CTRL
83 678 258 K562-S VOLASERTIB
92 264 765 K562-S DANUSERTIB
12 254 530 K562-R CTRL
50 306 258 K562-R VOLASERTIB
63 099 773 K562-R DANUSERTIB

CDK1

17 583 945 K562-S CTRL
27 577 747 K562-S VOLASERTIB
65 279 212 K562-S DANUSERTIB
17 346 359 K562-R CTRL
52 801 593 K562-R VOLASERTIB
46 512 714 K562-R DANUSERTIB

P-CDK1(Y15)

15 769 459 K562-S CTRL
34 352 915 K562-S VOLASERTI
66 190 409 K562-S DANUSERTI
11 555 731 K562-R CTRL
22 709 874 K562-R VOLASERTI
84 870 744 K562-R DANUSERTI

BETA ACTIN

50 120 924 K562-S CTRL
59 254 359 K562-S VOLASERTI
61 766 702 K562-S DANUSERTI
67 151 803 K562-R CTRL
67 312 832 K562-R VOLASERTI
77 311 409 K562-R DANUSERTI

FIGURE 4 C

P-H2AX(Y143)		BETA ACTIN	
0	CTRL	55 797 116	CTRL
358 263	AZD1775	56 712 894	AZD1775
14 661 276	DANUSERTIB	54 793 045	DANUSERTIB
1 828 163	VOLASERTIB	44 687 066	VOLASERTIB
14 935 376	A+D	48 366 258	A+D
30 962 326	A+V	47 880 773	A+V

FIGURE 4 D

P-H2AX(Y143)		BETA ACTIN	
0	CTRL	22 276 589	CTRL
14 786 033	AZD1775	23 464 983	AZD1775
3 613 255	DANUSERTIB	18 663 811	DANUSERTIB
6 106 033	VOLASERTIB	21 709 397	VOLASERTIB
19 163 225	A+D	18 244 004	A+D
27 656 276	A+V	20 273 154	A+V

FIGURE 5 C

WEE1		UNCLEAVED PARP	
22 371 832	CTRL	21 045 054	CTRL
22 792 903	AZD1775	188 435	AZD1775
26 062 539	DANUSERTIB	7 391 276	DANUSERTIB
30 423 489	VOLASERTIB	1 748 497	VOLASERTIB
13 422 246	A+D	18 762 317	A+D
16 963 175	A+V	12 920 832	A+V
P-WEE(S642)		CLEAVED PARP	
3 971 154	CTRL	0	CTRL
0	AZD1775	3 914 397	AZD1775
11 862 347	DANUSERTIB	27 933 397	DANUSERTIB
28 117 953	VOLASERTIB	5 829 761	VOLASERTIB
0	A+D	30 959 518	A+D
0	A+V	25 685 782	A+V
AURORA KINASE A		UNCLEAVED CASPASE-9	
24 501 024	CTRL	21 241 246	CTRL
15 752 418	AZD1775	22 080 903	AZD1775
8 607 397	DANUSERTIB	19 787 075	DANUSERTIB
7 641 054	VOLASERTIB	27 474 518	VOLASERTIB
6 401 640	A+D	29 669 761	A+D
6 692 296	A+V	12 655 882	A+V
P-AURORA KINASE A (T288)		CLEAVED CASPASE-9	
22 077 761	CTRL	0	CTRL
28 516 660	AZD1775	19 011 865	AZD1775
2 273 891	DANUSERTIB	36 788 940	DANUSERTIB
2 089 841	VOLASERTIB	6 806 953	VOLASERTIB
1 518 234	A+D	19 313 953	A+D
1 890 184	A+V	11 073 459	A+V

FIGURE 5 C

UNCLEAVED CASPASE-3

14 534 004	CTRL
4 848 225	AZD1775
33 920 075	DANUSERTIB
26 439 882	VOLASERTIB
4 334 347	A+D
25 482 368	A+V

CLEAVED CASPASE-3

0	CTRL
31 367 447	AZD1775
32 510 891	DANUSERTIB
33 143 083	VOLASERTIB
9 047 054	A+D
15 119 104	A+V

BAX

0	CTRL
17 951 468	AZD1775
4 003 690	DANUSERTIB
7 340 397	VOLASERTIB
27 195 782	A+D
23 596 832	A+V

BETA ACTIN

30 731 933	CTRL
34 971 640	AZD1775
33 398 347	DANUSERTIB
28 138 296	VOLASERTIB
31 863 690	A+D
35 261 589	A+V

FIGURE 5 D

WEE1

15 594 953	CTRL
9 475 125	AZD1775
30 351 125	DANUSERTIB
24 187 832	VOLASERTIB
13 856 468	A+D
13 318 276	A+V

P-WEE(S642)

8 882 832	CTRL
832 669	AZD1775
23 177 953	DANUSERTIB
5 728 296	VOLASERTIB
0	A+D
0	A+V

AURORA KINASE A

22 466 368	CTRL
14 958 016	AZD1775
13 194 359	DANUSERTIB
6 899 489	VOLASERTIB
6 475 861	A+D
5 388 640	A+V

P-AURORA KINASE A (T288)

17 291 317	CTRL
26 958 711	AZD1775
784 305	DANUSERTIB
2 239 376	VOLASERTIB
1 906 891	A+D
1 330 598	A+V

UNCLEAVED PARP

18 935 246	CTRL
23 176 489	AZD1775
17 407 288	DANUSERTIB
11 198 033	VOLASERTIB
17 875 054	A+D
0	A+V

CLEAVED PARP

0	CTRL
27 570 861	AZD1775
9 455 569	DANUSERTIB
19 485 225	VOLASERTIB
27 587 589	A+D
12 531 075	A+V

UNCLEAVED CASPASE-9

9 612 054	CTRL
9 373 276	AZD1775
21 307 640	DANUSERTIB
23 506 761	VOLASERTIB
29 102 619	A+D
12 507 518	A+V

CLEAVED CASPASE-9

977 820	CTRL
11 959 953	AZD1775
21 000 548	DANUSERTIB
25 473 033	VOLASERTIB
28 925 912	A+D
14 656 296	A+V

FIGURE 5 D

UNCLEAVED CASPASE-3	BAX
10 225 761	0
29 642 861	5 943 861
19 323 690	2 887 447
32 231 012	19 151 175
26 028 497	14 600 246
11 847 761	10 170 761
CLEAVED CASPASE-3	BETA ACTIN
0	29 459 861
19 180 418	23 638 497
15 670 761	17 911 397
21 535 004	20 650 033
7 657 569	22 424 983
24 298 832	20 119 569
